# Crystal structures of (12*E*)-12-(4-benzyl­idene)-7,7,16-trimethyl-3-(4-methyl­phen­yl)-1-oxa-16-aza­tetra­cyclo­[11.2.1.0^2,11^.0^4,9^]hexa­deca-2(11),4(9)-dien-5-one and (12*E*)-12-(4-bromo­benzyl­idene)-73-(4-bromo­phen­yl)-,7,16-trimethyl-10-oxa-16-aza­tetra­cyclo­[11.2.1.0^2,11^.0^4,9^]hexa­deca-2(11),4(9)-dien-5-one

**DOI:** 10.1107/S205698902300275X

**Published:** 2023-03-28

**Authors:** C. Selva Meenatchi, R. Vishnupriya, J. Suresh, S. Raja Rubina, S. Selvanayagam, S. R. Bhandari

**Affiliations:** a Department of Physics, The Madura College, Madurai 625 011, India; b Department of Organic Chemistry, School of Chemistry, Madurai Kamaraj University, Madurai 625 021, India; c PG & Research Department of Physics, Government Arts College, Melur 625 106, India; d Department of Physics, Bhairahawa M. Campus, Tribhuvan University, Nepal; Venezuelan Institute of Scientific Research, Venezuela

**Keywords:** crystal structure, aza­bicyclo derivatives, superposition, C—H⋯O intra­molecular inter­actions, C—H⋯O inter­molecular hydrogen bonds, Hirshfeld surface analysis

## Abstract

The title compounds, C_32_H_35_NO_2_, (I), and C_30_H_29_Br_2_NO_2_, (II), differ by the presence of a bromine atom instead of a methyl atom in the *para* position of two benzene rings of compound (II). The two compounds have a structural overlap r.m.s. deviation of 0.27 Å. The pyran and seven-membered cyclo­heptene rings in both structures adopt boat and boat-sofa conformations, respectively. Intra- and inter­molecular C—H⋯O hydrogen bonds are responsible for the consolidation of the crystal packing of both mol­ecules. In addition to this, weak C—H⋯π inter­actions are also observed. The inter­molecular inter­actions were qu­anti­fied and analysed using Hirshfeld surface analysis.

## Abstract 2

The title compounds, C_32_H_35_NO_2_, (I), and C_30_H_29_Br_2_NO_2_, (II), differ by the presence of a bromine atom instead of a methyl atom in the *para* position of two phenyl rings of compound (II), with an r.m.s. deviation of 0.27 Å between these compounds.

## 1. Chemical context

The tropane skeleton is found widely in both natural and manufactured medications. It is the fundamental component of many beneficial alkaloids, including atropine, scopolamine, and cocaine, whose derivatives are important in the treatment of neurological and psychiatric conditions such depression and panic disorder (Cheenpracha *et al.*, 2013 ▸; Afewerki *et al.*, 2019 ▸; Dongbang *et al.*, 2021 ▸). It is also a key component in the synthesis of newer types of drugs. Tropane derivatives are used to treat irritable bowel syndrome, peptic ulcers, colic, cystitis, and pancreatitis thanks to their anti-spasmodic properties. In view of the above importance, we have undertaken a single-crystal X-ray diffraction study for the title compounds, and the results are presented herein. 
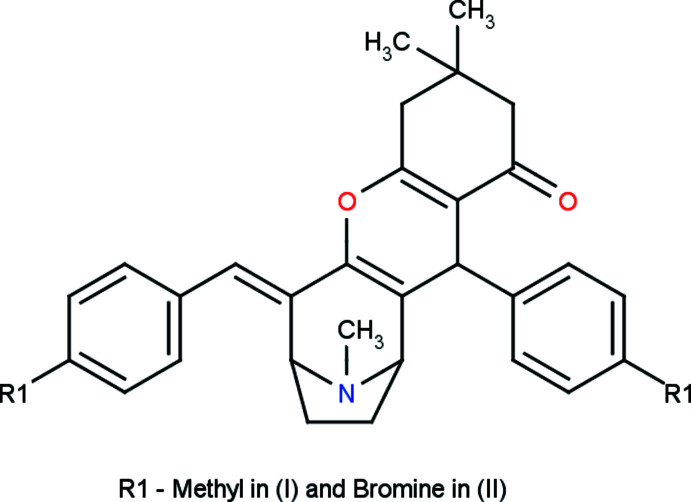


## 2. Structural commentary

**Figure 1:**
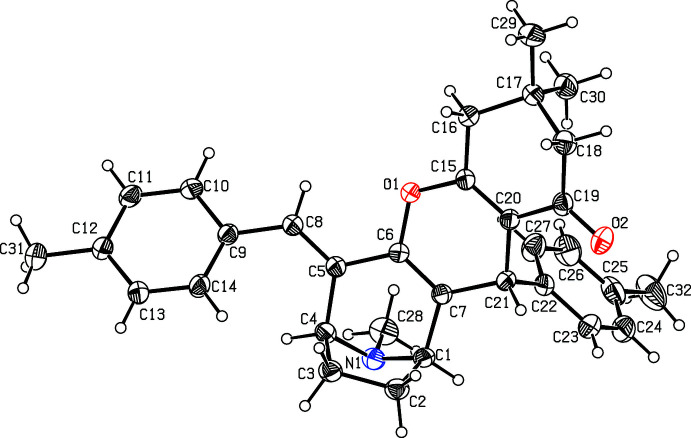
A view of the mol­ecular structure of compound (I), showing the atom labelling. Displacement ellipsoids are drawn at the 30% probability level.

**Figure 3:**
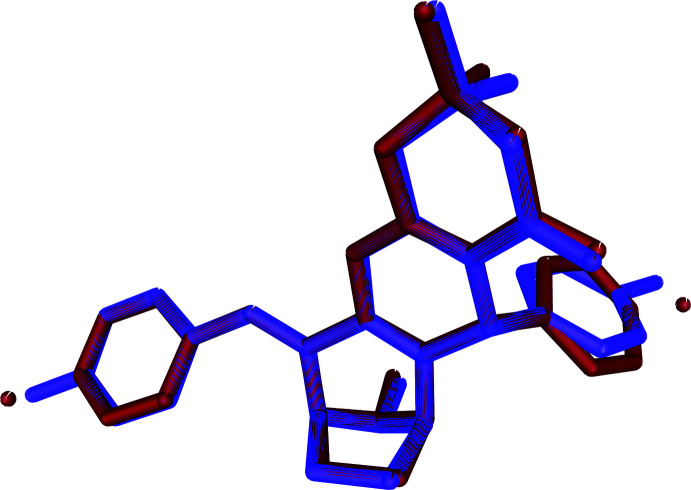
Superposition of compound (I) (violet) and compound (II) (brown) except for atoms Br1, Br2 and C21.

The mol­ecular structure of the title compounds (I) and (II) are illustrated in Figs. 1 ▸ and 2 ▸, respectively. Fig. 3 ▸ shows the superposition of the two compounds except for atom C21 using *Qmol* (Gans & Shalloway, 2001 ▸); the r.m.s. deviation is 0.27 Å. The methyl­phenyl rings in (I) are oriented at a dihedral angle of 57.7 (1)°. The methyl atoms C31 and C32 in (I) deviate by −0.036 (1) and 0.053 (1) Å, respectively, from the rings to which they are attached. The bromo­phenyl rings in (II) are oriented at a dihedral angle of 54.3 (1)°. Bromine atoms Br1 and Br2 deviate by 0.050 (1) and 0.037 (1) Å, respectively, from the rings to which they are attached.

The seven-membered ring (C1–C7) in both compounds has a boat-sofa conformation, with puckering parameters (Boessenkool & Boeyens, 1980 ▸) *q*_2_ = 1.021 (2) and *q*_3_ = 0.391 (2) Å in (I) and *q*_2_ = 1.053 (2) and *q*_3_ = 0.374 (2) Å in (II). The piperidine ring (N1/C4–C7/C1) has a half-boat conformation in both compounds, with atoms C4–C7/C1 in the plane and atom N1 deviating by −0.793 (1) [in (I)] or 0.785 (1) Å [in (II)] from this plane. The pyran ring (C6/O1/C15/C20/C21/C7) adopts boat conformation in both structures with puckering parameters (Cremer & Pople, 1975 ▸) *q*_2_ = *Q*_T_ = 0.185 (2) Å and φ = 43.9 (1)° [in (I)] and *q*_2_ = *Q*_T_ = 0.087 (1) Å and φ = 47.1 (1)° [in (II)]. The cyclo­hexene ring (C15–C20) has a distorted sofa conformation in both (I) and (II), with Δ*C_s_* (C17) asymmetry parameters (Nardelli, 1983 ▸) of 0.058 (1) and 0.004 (1), respectively.

Intra­molecular C—H⋯O hydrogen bonds are observed (Tables 1 ▸ and 2 ▸)

## 3. Supra­molecular features

**Figure 4:**
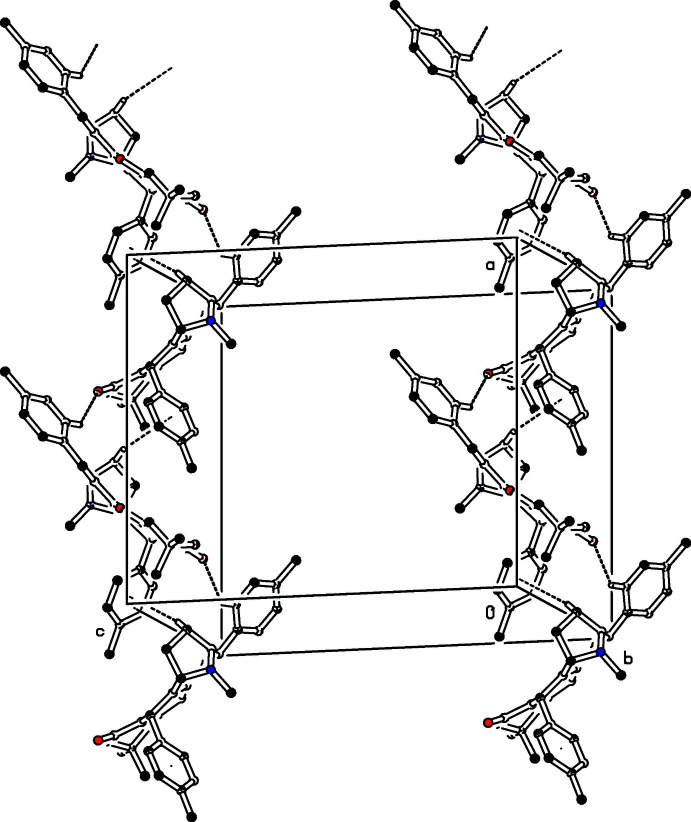
The crystal packing of the title compound (I) viewed along *b* axis. The C—H⋯O and C—H⋯π inter­molecular inter­actions are shown as dashed lines. For clarity, H atoms not involved in these inter­actions have been omitted.

In the crystal of (I), mol­ecules associate *via* C—H⋯O inter­molecular inter­actions (C14—H14⋯O2^i^, Table 1 ▸), forming chains propagating along the [100] direction, see Fig. 4 ▸. In addition to this, inversion-related mol­ecules are linked into chains by C—H⋯π inter­actions [C3—H3*B*⋯*Cg*, where *Cg* is the centroid of the C22–C27 benzene ring of the symmetry-related mol­ecule at (− 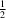 + *x*, 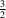 − *y*, −*z*), see Fig. 4 ▸]. These two inter­actions form chains running in a helical manner along [101].

**Figure 5:**
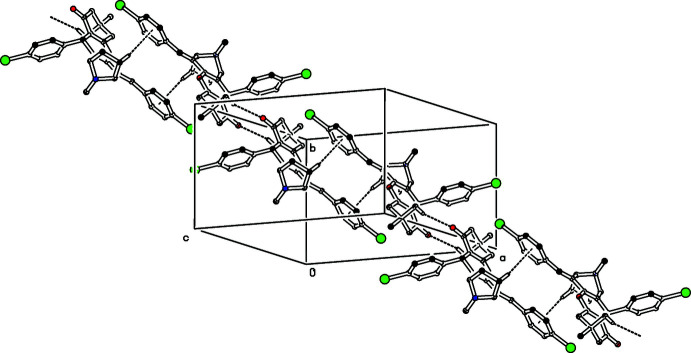
The centrosymmetrical dimer formed in compound (II) *via* C—H⋯O hydrogen bonds (dashed lines). The dimers are linked by C—H⋯π inter­actions (dashed lines). For clarity H atoms, not involved in these inter­actions have been omitted.

In the crystal of (II), mol­ecules are also linked *via* C—H⋯O inter­molecular inter­action (C30—H30*A*⋯O2^i^, Table 2 ▸), here forming centrosymmetrical dimers with an 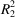 (12) ring motif (Fig. 5 ▸). The dimers are further linked into chains by C—H⋯π inter­actions (C3—H3*B*⋯*Cg*, where *Cg* is the centroid of the C9–C14 benzene ring of the symmetry-related mol­ecule at (2 − *x*, 2 − *y*, −*z*), see Fig. 4 ▸]. These two inter­actions form chains running diagonally along [110].

## 4. Hirshfeld surface analysis

**Figure 6:**
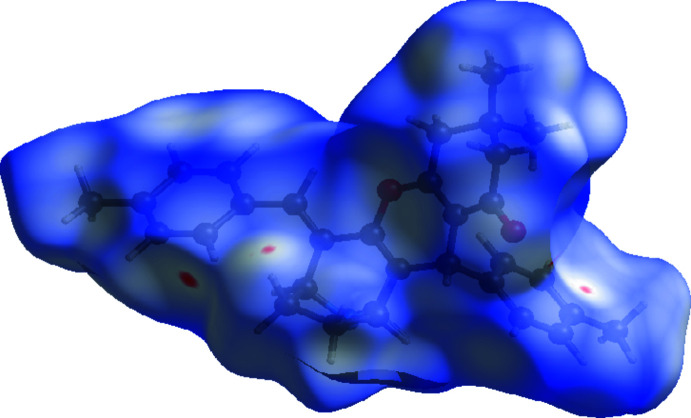
A view of the Hirshfeld surface mapped over *d*_norm_ in the range −0.0701 to +1.6693 arbitrary units for compound (I).

To further characterize the inter­molecular inter­actions in the title compound, we carried out a Hirshfeld surface (HS) analysis (Spackman & Jayatilaka, 2009 ▸) using *Crystal Explorer 21* (Spackman *et al.*, 2021 ▸) and generated the associated two dimensional fingerprint plots (McKinnon *et al.*, 2007 ▸). The HS mapped over *d*_norm_ in the range −0.0701 to +1.6693 a.u. for compound (I) and −0.1162 to +1.5964 a.u. for compound (II) are illustrated in Figs. 6 ▸ and 7 ▸, using colours to indicate contacts that are shorter (red areas), equal to (white areas), or longer than (blue areas) the sum of the van der Waals radii (Ashfaq *et al.*, 2021 ▸).

**Figure 8:**
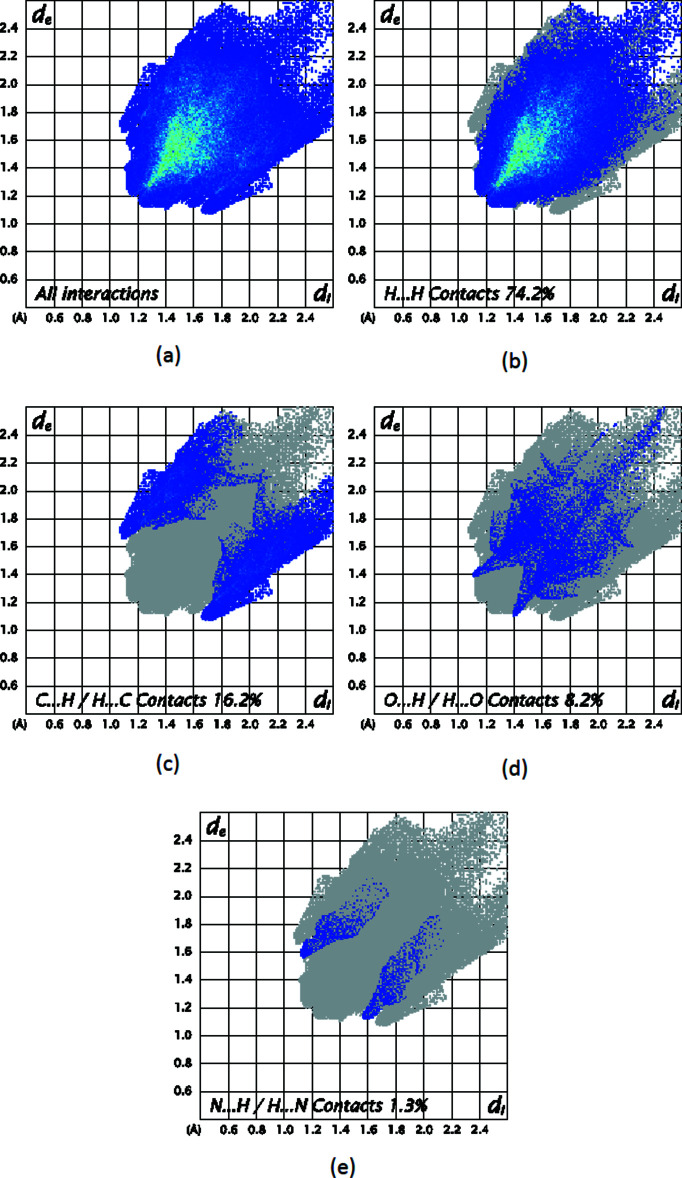
Two-dimensional fingerprint plots for compound (I), showing (*a*) all inter­actions, and delineated into (*b*) H⋯H, (*c*) C⋯H/H⋯C, (*d*) O⋯H/H⋯O and (*e*) N⋯H/H⋯N inter­actions. The *d*_i_ and *d*_e_ values are the closest inter­nal and external distances (in Å) from given points on the Hirshfeld surface.

**Figure 9:**
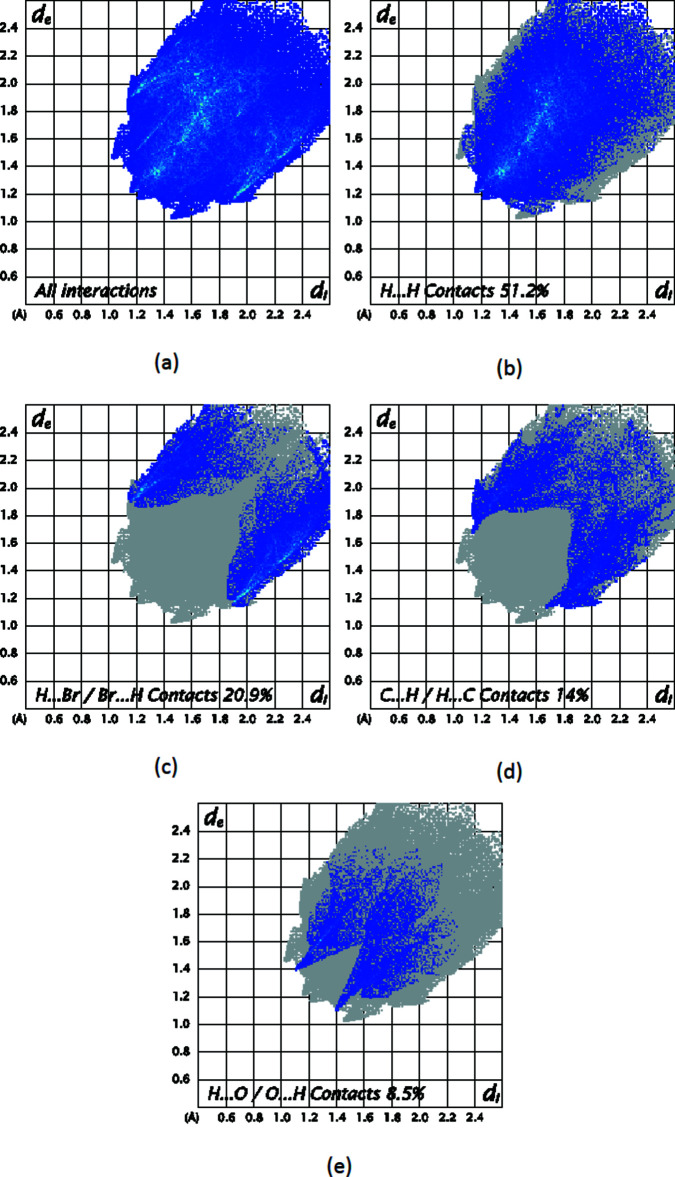
Two-dimensional fingerprint plots for compound (II), showing (*a*) all inter­actions, and delineated into (*b*) H⋯H, (*c*) Br⋯H/H⋯Br, (*d*) C⋯H/H⋯C and (*e*) O⋯H/H⋯O inter­actions. The *d*_i_ and *d*_e_ values are the closest inter­nal and external distances (in Å) from given points on the Hirshfeld surface.

The two-dimensional fingerprint plots provide qu­anti­tative information about the non-covalent inter­actions and the crystal packing in terms of the percentage contribution of the inter­atomic contacts (Spackman & McKinnon, 2002 ▸; Ashfaq *et al.*, 2021 ▸). The HS analysis reveals that H⋯H (74.2%) and H⋯C/C⋯H (16.2%) contacts are the main contributors to the crystal packing, followed by O⋯H/H⋯O (8.2%) and N⋯H/H⋯N (1.3%) contacts for compound (I) (Fig. 8 ▸). In compound (II), H⋯H (51.2%) and H⋯Br/Br⋯H (20.9%) contacts are the main contributors to the crystal packing, followed by C⋯H/H⋯C (14%) and O⋯H/H⋯O (8.5%) contacts (Fig. 9 ▸). The fragment patches on the HS provide an easy way to investigate the nearest neighbour coordination environment of a mol­ecule (coordination number), which is 14 for compound (I) and 15 for compound (II).

## 5. Synthesis and crystallization

Compound (I) was synthesized from a mixture of 8-methyl-8-aza­bicyclo­[3.2.1]octan-3-one and two equivalents of 4-methyl­benzaldehyde and 5,5-di­methyl­cyclo­hexane-1,3-dione dissolved in ethanol/acetic acid and refluxed for 12 h. After completion of the reaction, as indicated by thin layer chromatography (TLC), the mixture was cooled to room temperature, poured into ice-cold water and neutralized with a saturated solution of sodium bicarbonate. The compound was further recrystallized from ethanol to obtain crystals suitable for single crystal X-ray analysis.

Compound (II) was synthesized from a mixture of 8-methyl-8-aza­bicyclo­[3.2.1]octan-3-one, two equivalents of 4-bromo­benzaldehyde and 5,5-di­methyl­cyclo­hexane-1,3-dione dis­solved in ethanol/acetic acid and refluxed for 12 h. After completion of the reaction, as indicated by thin layer chromatography (TLC), the mixture was cooled to room temperature, poured into ice-cold water and neutralized with a saturated solution of sodium bicarbonate. The compound was further recrystallized from ethanol to obtain crystals suitable for single crystal X-ray analysis.

## 6. Refinement

Crystal data, data collection and structure refinement details are summarized in Table 3 ▸. In both (I) and (II), H atoms were placed in idealized positions and allowed to ride on their parent atoms: C—H = 0.93–0.98 Å, with *U*_iso_(H) = 1.5*U*_eq_(C-meth­yl) and 1.2*U*_eq_(C) for other H atoms.

## Supplementary Material

Crystal structure: contains datablock(s) I, II, global. DOI: 10.1107/S205698902300275X/zn2026sup1.cif

Structure factors: contains datablock(s) I. DOI: 10.1107/S205698902300275X/zn2026Isup2.hkl

Structure factors: contains datablock(s) II. DOI: 10.1107/S205698902300275X/zn2026IIsup3.hkl

CCDC references: 2251053, 2251052

Additional supporting information:  crystallographic information; 3D view; checkCIF report

## Figures and Tables

**Figure 2:**
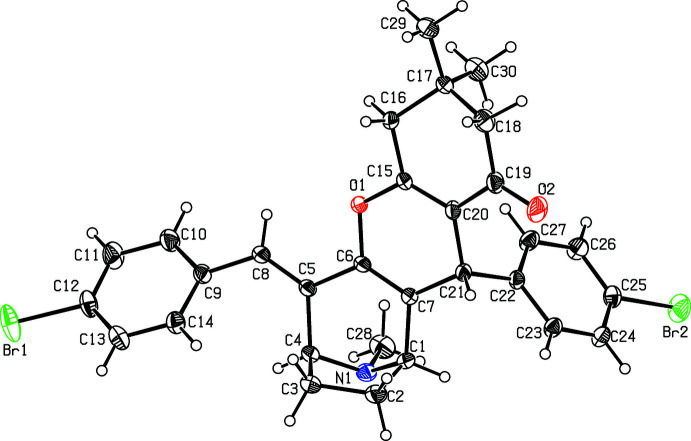
A view of the mol­ecular structure of compound (II), showing the atom labelling. Displacement ellipsoids are drawn at the 30% probability level.

**Figure 7:**
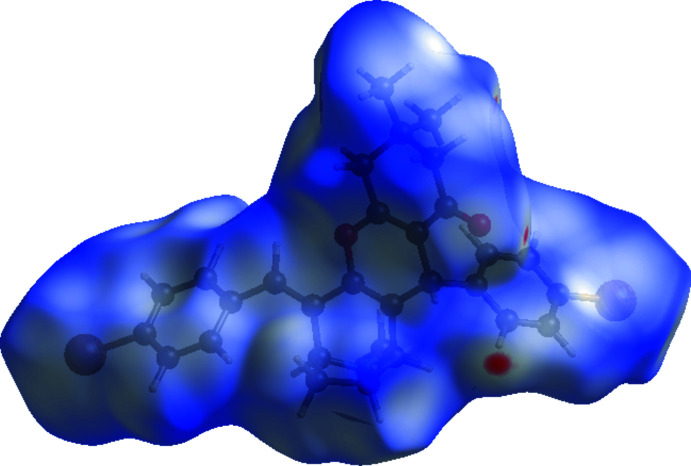
A view of the Hirshfeld surface mapped over *d*_norm_ in the range −0.1162 to +1.5964 arbitrary units for compound (II).

**Table 1:** Hydrogen-bond geometry (Å, °) for (I) *Cg* is the centroid of the C22–C27 ring.

*D*—H⋯*A*	*D*—H	H⋯*A*	*D*⋯*A*	*D*—H⋯*A*
C8—H8⋯O1	0.93	2.37	2.766 (2)	105
C14—H14⋯O2^i^	0.93	2.59	3.285 (2)	131
C3—H3*B*⋯*Cg* ^i^	0.97	2.88	3.795 (2)	152

Symmetry code: (i) 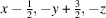 .

**Table 2:** Hydrogen-bond geometry (Å, °) for (II) *Cg* is the centroid of the C9–C14 ring.

*D*—H⋯*A*	*D*—H	H⋯*A*	*D*⋯*A*	*D*—H⋯*A*
C8—H8⋯O1	0.93	2.36	2.742 (3)	105
C30—H30*A*⋯O2^i^	0.96	2.58	3.306 (5)	132
C3—H3*B*⋯*Cg* ^ii^	0.97	2.84	3.798 (3)	169

Symmetry codes: (i) 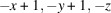 ; (ii) 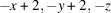 .

**Table 3:** Experimental details

	(I)	(II)
Crystal data		
Chemical formula	C_32_H_35_NO_2_	C_30_H_29_Br_2_NO_2_
*M*_r_	465.61	595.36
Crystal system, space group	Orthorhombic, *P* *b* *c* *a*	Monoclinic, *P*2_1_/*n*
Temperature (K)	293	293
*a*, *b*, *c* (Å)	16.7576 (6), 16.3223 (7), 19.2416 (8)	16.051 (3), 9.7793 (17), 17.005 (3)
α, β, γ (°)	90, 90, 90	90, 96.312 (5), 90
*V* (Å^3^)	5263.0 (4)	2653.1 (8)
*Z*	8	4
Radiation type	Mo *K*α	Mo *K*α
μ (mm^−1^)	0.07	3.08
Crystal size (mm)	0.21 × 0.18 × 0.16	0.20 × 0.17 × 0.15

Data collection		
Diffractometer	Bruker SMART APEX CCD area-detector	Bruker SMART APEX CCD area-detector
No. of measured, independent and observed [*I* > 2σ(*I*)] reflections	186331, 8013, 5206	133143, 6556, 4933
*R*_int_	0.097	0.091
(sin θ/λ)_max_ (Å^−1^)	0.714	0.667

Refinement		
*R*[*F* ^2^ > 2σ(*F* ^2^)], *wR*(*F* ^2^), *S*	0.059, 0.213, 1.04	0.050, 0.141, 1.09
No. of reflections	8013	6556
No. of parameters	319	316
H-atom treatment	H-atom parameters constrained	H-atom parameters constrained
Δρ_max_, Δρ_min_ (e Å^−3^)	0.31, −0.23	0.54, −0.71

Computer programs: *APEX2* and *SAINT* (Bruker, 2009 ▸), *SHELXT2018/2* (Sheldrick, 2015*a* ▸), *SHELXL2018/3* (Sheldrick, 2015*b* ▸), *ORTEP-3 for Windows* (Farrugia, 2012 ▸) and *PLATON* (Spek, 2020 ▸).

## supplementary crystallographic information

### (12E)-12-(4-Benzylidene)-7,7,16-trimethyl-3-(4-methylphenyl)-1-oxa-16-azatetracyclo[11.2.1.02,11.04,9]hexadeca-2(11),4(9)-dien-5-one (I) Crystal data

**Table:**

C_32_H_35_NO_2_	*D*_x_ = 1.175 Mg m^−^^3^
*M**_r_* = 465.61	Mo *K*α radiation, λ = 0.71073 Å
Orthorhombic, *P**b**c**a*	Cell parameters from 118400 reflections
*a* = 16.7576 (6) Å	θ = 3.1–27.8°
*b* = 16.3223 (7) Å	µ = 0.07 mm^−^^1^
*c* = 19.2416 (8) Å	*T* = 293 K
*V* = 5263.0 (4) Å^3^	Block, colourless
*Z* = 8	0.21 × 0.18 × 0.16 mm
*F*(000) = 2000	

### (12E)-12-(4-Benzylidene)-7,7,16-trimethyl-3-(4-methylphenyl)-1-oxa-16-azatetracyclo[11.2.1.02,11.04,9]hexadeca-2(11),4(9)-dien-5-one (I) Data collection

**Table:**

Bruker SMART APEX CCD area-detector diffractometer	*R*_int_ = 0.097
Radiation source: fine-focus sealed tube	θ_max_ = 30.5°, θ_min_ = 2.9°
ω and φ scans	*h* = −23→23
186331 measured reflections	*k* = −23→23
8013 independent reflections	*l* = −27→27
5206 reflections with *I* > 2σ(*I*)	

### (12E)-12-(4-Benzylidene)-7,7,16-trimethyl-3-(4-methylphenyl)-1-oxa-16-azatetracyclo[11.2.1.02,11.04,9]hexadeca-2(11),4(9)-dien-5-one (I) Refinement

**Table:**

Refinement on *F*^2^	Hydrogen site location: inferred from neighbouring sites
Least-squares matrix: full	H-atom parameters constrained
*R*[*F*^2^ > 2σ(*F*^2^)] = 0.059	*w* = 1/[σ^2^(*F*_o_^2^) + (0.1059*P*)^2^ + 1.8765*P*] where *P* = (*F*_o_^2^ + 2*F*_c_^2^)/3
*wR*(*F*^2^) = 0.213	(Δ/σ)_max_ < 0.001
*S* = 1.04	Δρ_max_ = 0.31 e Å^−^^3^
8013 reflections	Δρ_min_ = −0.23 e Å^−^^3^
319 parameters	Extinction correction: *SHELXL2018/3* (Sheldrick 2015b), Fc^*^=kFc[1+0.001xFc^2^λ^3^/sin(2θ)]^-1/4^
0 restraints	Extinction coefficient: 0.0063 (9)

### (12E)-12-(4-Benzylidene)-7,7,16-trimethyl-3-(4-methylphenyl)-1-oxa-16-azatetracyclo[11.2.1.02,11.04,9]hexadeca-2(11),4(9)-dien-5-one (I) Special details

**Table:**

Geometry. All esds (except the esd in the dihedral angle between two l.s. planes) are estimated using the full covariance matrix. The cell esds are taken into account individually in the estimation of esds in distances, angles and torsion angles; correlations between esds in cell parameters are only used when they are defined by crystal symmetry. An approximate (isotropic) treatment of cell esds is used for estimating esds involving l.s. planes.

### (12E)-12-(4-Benzylidene)-7,7,16-trimethyl-3-(4-methylphenyl)-1-oxa-16-azatetracyclo[11.2.1.02,11.04,9]hexadeca-2(11),4(9)-dien-5-one (I) Fractional atomic coordinates and isotropic or equivalent isotropic displacement parameters (Å^2^)

**Table:**

	*x*	*y*	*z*	*U*_iso_*/*U*_eq_	
O1	0.21074 (7)	0.58367 (7)	0.03767 (7)	0.0427 (3)	
O2	0.34366 (9)	0.72866 (9)	−0.13951 (8)	0.0577 (4)	
N1	0.16512 (8)	0.78470 (9)	0.16257 (8)	0.0424 (3)	
C1	0.18856 (10)	0.80464 (10)	0.09126 (10)	0.0405 (4)	
H1	0.228435	0.848551	0.090424	0.049*	
C2	0.10849 (11)	0.83394 (11)	0.06103 (11)	0.0463 (4)	
H2A	0.105807	0.823989	0.011381	0.056*	
H2B	0.100310	0.891866	0.069747	0.056*	
C3	0.04652 (10)	0.78136 (11)	0.10060 (11)	0.0459 (4)	
H3A	0.009062	0.815815	0.125555	0.055*	
H3B	0.017141	0.746445	0.068828	0.055*	
C4	0.09680 (10)	0.72978 (10)	0.15142 (9)	0.0400 (4)	
H4	0.067771	0.720868	0.194982	0.048*	
C5	0.12141 (9)	0.64808 (10)	0.11912 (9)	0.0370 (3)	
C6	0.18632 (9)	0.65699 (9)	0.06908 (9)	0.0371 (3)	
C7	0.21783 (9)	0.72967 (10)	0.05213 (9)	0.0376 (3)	
C8	0.08674 (10)	0.57522 (10)	0.13228 (9)	0.0396 (4)	
H8	0.109249	0.529878	0.110539	0.048*	
C9	0.01749 (10)	0.55924 (10)	0.17700 (9)	0.0382 (3)	
C10	0.01149 (11)	0.48435 (10)	0.21179 (10)	0.0435 (4)	
H10	0.050998	0.445040	0.205445	0.052*	
C11	−0.05206 (12)	0.46743 (11)	0.25556 (10)	0.0475 (4)	
H11	−0.053642	0.417504	0.278801	0.057*	
C12	−0.11325 (11)	0.52321 (11)	0.26546 (10)	0.0452 (4)	
C13	−0.10909 (12)	0.59637 (12)	0.22870 (11)	0.0511 (5)	
H13	−0.149884	0.634551	0.233678	0.061*	
C14	−0.04585 (11)	0.61396 (11)	0.18485 (11)	0.0486 (4)	
H14	−0.045578	0.663009	0.160261	0.058*	
C15	0.25631 (10)	0.59020 (10)	−0.02083 (9)	0.0377 (3)	
C16	0.26745 (11)	0.50897 (11)	−0.05506 (10)	0.0447 (4)	
H16A	0.218260	0.493661	−0.078242	0.054*	
H16B	0.278380	0.468083	−0.019734	0.054*	
C17	0.33577 (11)	0.50880 (11)	−0.10836 (10)	0.0459 (4)	
C18	0.32508 (13)	0.58496 (12)	−0.15407 (10)	0.0511 (4)	
H18A	0.369117	0.587793	−0.186698	0.061*	
H18B	0.276320	0.579014	−0.180759	0.061*	
C19	0.32134 (10)	0.66405 (11)	−0.11423 (9)	0.0420 (4)	
C20	0.28567 (9)	0.66154 (10)	−0.04453 (9)	0.0368 (3)	
C21	0.28068 (9)	0.73973 (10)	−0.00295 (9)	0.0386 (3)	
H21	0.264501	0.784237	−0.034095	0.046*	
C22	0.36107 (10)	0.76184 (11)	0.02941 (10)	0.0418 (4)	
C23	0.39866 (12)	0.83551 (12)	0.01558 (13)	0.0555 (5)	
H23	0.375068	0.873040	−0.014421	0.067*	
C24	0.47229 (14)	0.85379 (15)	0.04666 (15)	0.0689 (7)	
H24	0.496623	0.903724	0.037033	0.083*	
C25	0.50927 (12)	0.79995 (16)	0.09086 (13)	0.0624 (6)	
C26	0.47107 (13)	0.72732 (17)	0.10501 (14)	0.0688 (6)	
H26	0.494539	0.690036	0.135313	0.083*	
C27	0.39826 (12)	0.70872 (14)	0.07493 (13)	0.0596 (5)	
H27	0.373750	0.659182	0.085642	0.071*	
C28	0.22827 (13)	0.75329 (15)	0.20727 (12)	0.0588 (5)	
H28A	0.206981	0.742153	0.252617	0.088*	
H28B	0.249391	0.703701	0.187734	0.088*	
H28C	0.270044	0.793302	0.210916	0.088*	
C29	0.33113 (16)	0.43051 (14)	−0.15184 (14)	0.0669 (6)	
H29A	0.337967	0.383735	−0.122184	0.100*	
H29B	0.280025	0.427542	−0.174248	0.100*	
H29C	0.372469	0.431112	−0.186370	0.100*	
C30	0.41730 (12)	0.51242 (14)	−0.07213 (13)	0.0590 (5)	
H30A	0.420629	0.561454	−0.044726	0.088*	
H30B	0.423397	0.465485	−0.042538	0.088*	
H30C	0.458865	0.512600	−0.106463	0.088*	
C31	−0.18224 (15)	0.50520 (14)	0.31352 (13)	0.0645 (6)	
H31A	−0.198385	0.449151	0.307978	0.097*	
H31B	−0.226148	0.540633	0.302414	0.097*	
H31C	−0.166058	0.514370	0.360747	0.097*	
C32	0.59000 (14)	0.8188 (2)	0.12213 (18)	0.0941 (10)	
H32A	0.605958	0.873155	0.109106	0.141*	
H32B	0.628525	0.780055	0.105291	0.141*	
H32C	0.586766	0.815138	0.171847	0.141*	

### (12E)-12-(4-Benzylidene)-7,7,16-trimethyl-3-(4-methylphenyl)-1-oxa-16-azatetracyclo[11.2.1.02,11.04,9]hexadeca-2(11),4(9)-dien-5-one (I) Atomic displacement parameters (Å^2^)

**Table:**

	*U*^11^	*U*^22^	*U*^33^	*U*^12^	*U*^13^	*U*^23^
O1	0.0463 (6)	0.0325 (6)	0.0492 (7)	−0.0009 (5)	0.0145 (5)	0.0013 (5)
O2	0.0641 (9)	0.0546 (8)	0.0545 (8)	−0.0103 (6)	0.0075 (7)	0.0150 (6)
N1	0.0379 (7)	0.0422 (8)	0.0471 (8)	0.0005 (6)	−0.0033 (6)	−0.0055 (6)
C1	0.0366 (7)	0.0319 (7)	0.0530 (10)	−0.0008 (6)	0.0002 (7)	−0.0028 (7)
C2	0.0473 (9)	0.0391 (9)	0.0525 (10)	0.0060 (7)	−0.0018 (8)	0.0024 (7)
C3	0.0367 (8)	0.0425 (9)	0.0585 (11)	0.0037 (7)	−0.0035 (7)	−0.0012 (8)
C4	0.0364 (7)	0.0396 (8)	0.0442 (9)	0.0021 (6)	0.0024 (6)	−0.0032 (7)
C5	0.0334 (7)	0.0377 (8)	0.0400 (8)	0.0012 (6)	0.0020 (6)	0.0010 (6)
C6	0.0361 (7)	0.0327 (7)	0.0424 (8)	0.0013 (6)	0.0035 (6)	0.0000 (6)
C7	0.0323 (7)	0.0346 (8)	0.0460 (9)	−0.0001 (6)	0.0003 (6)	−0.0004 (6)
C8	0.0377 (8)	0.0374 (8)	0.0437 (9)	0.0020 (6)	0.0041 (6)	0.0028 (7)
C9	0.0391 (8)	0.0360 (8)	0.0395 (8)	−0.0014 (6)	0.0010 (6)	0.0042 (6)
C10	0.0477 (9)	0.0359 (8)	0.0468 (9)	0.0039 (7)	0.0027 (7)	0.0051 (7)
C11	0.0587 (11)	0.0376 (8)	0.0460 (10)	−0.0014 (8)	0.0041 (8)	0.0098 (7)
C12	0.0485 (9)	0.0413 (9)	0.0459 (10)	−0.0049 (7)	0.0073 (7)	0.0027 (7)
C13	0.0441 (9)	0.0442 (10)	0.0649 (12)	0.0032 (7)	0.0139 (8)	0.0083 (8)
C14	0.0420 (9)	0.0422 (9)	0.0617 (11)	0.0027 (7)	0.0073 (8)	0.0168 (8)
C15	0.0343 (7)	0.0379 (8)	0.0410 (8)	0.0003 (6)	0.0052 (6)	0.0025 (6)
C16	0.0452 (9)	0.0362 (8)	0.0526 (10)	−0.0033 (7)	0.0095 (8)	−0.0028 (7)
C17	0.0479 (9)	0.0410 (9)	0.0489 (10)	0.0037 (7)	0.0111 (8)	0.0010 (7)
C18	0.0616 (11)	0.0527 (11)	0.0390 (9)	0.0050 (9)	0.0069 (8)	0.0044 (8)
C19	0.0353 (7)	0.0464 (9)	0.0442 (9)	0.0007 (7)	0.0010 (6)	0.0084 (7)
C20	0.0337 (7)	0.0359 (8)	0.0409 (8)	0.0003 (6)	0.0019 (6)	0.0038 (6)
C21	0.0362 (7)	0.0322 (7)	0.0475 (9)	−0.0008 (6)	0.0039 (6)	0.0051 (6)
C22	0.0360 (8)	0.0395 (8)	0.0499 (9)	−0.0043 (6)	0.0069 (7)	−0.0033 (7)
C23	0.0500 (10)	0.0446 (10)	0.0720 (14)	−0.0095 (8)	0.0080 (9)	−0.0017 (9)
C24	0.0543 (12)	0.0584 (13)	0.0940 (18)	−0.0226 (10)	0.0181 (12)	−0.0237 (13)
C25	0.0369 (9)	0.0826 (15)	0.0677 (13)	−0.0041 (10)	0.0078 (9)	−0.0285 (12)
C26	0.0439 (10)	0.0877 (17)	0.0748 (15)	0.0026 (10)	−0.0091 (10)	0.0013 (13)
C27	0.0437 (10)	0.0609 (12)	0.0741 (14)	−0.0087 (9)	−0.0059 (9)	0.0121 (11)
C28	0.0528 (11)	0.0680 (13)	0.0557 (12)	0.0042 (10)	−0.0169 (9)	−0.0056 (10)
C29	0.0819 (16)	0.0496 (11)	0.0690 (14)	0.0036 (11)	0.0217 (12)	−0.0115 (10)
C30	0.0459 (10)	0.0580 (12)	0.0730 (14)	0.0081 (9)	0.0069 (9)	0.0132 (10)
C31	0.0683 (13)	0.0578 (12)	0.0674 (14)	−0.0054 (10)	0.0250 (11)	0.0097 (10)
C32	0.0405 (11)	0.129 (3)	0.112 (2)	−0.0109 (14)	0.0027 (13)	−0.055 (2)

### (12E)-12-(4-Benzylidene)-7,7,16-trimethyl-3-(4-methylphenyl)-1-oxa-16-azatetracyclo[11.2.1.02,11.04,9]hexadeca-2(11),4(9)-dien-5-one (I) Geometric parameters (Å, º)

**Table:**

O1—C15	1.364 (2)	C16—H16B	0.9700
O1—C6	1.4018 (19)	C17—C29	1.529 (3)
O2—C19	1.220 (2)	C17—C18	1.533 (3)
N1—C28	1.457 (2)	C17—C30	1.535 (3)
N1—C1	1.464 (2)	C18—C19	1.503 (3)
N1—C4	1.470 (2)	C18—H18A	0.9700
C1—C7	1.518 (2)	C18—H18B	0.9700
C1—C2	1.539 (2)	C19—C20	1.469 (2)
C1—H1	0.9800	C20—C21	1.509 (2)
C2—C3	1.547 (3)	C21—C22	1.527 (2)
C2—H2A	0.9700	C21—H21	0.9800
C2—H2B	0.9700	C22—C27	1.381 (3)
C3—C4	1.541 (2)	C22—C23	1.383 (2)
C3—H3A	0.9700	C23—C24	1.403 (3)
C3—H3B	0.9700	C23—H23	0.9300
C4—C5	1.528 (2)	C24—C25	1.371 (4)
C4—H4	0.9800	C24—H24	0.9300
C5—C8	1.348 (2)	C25—C26	1.375 (4)
C5—C6	1.460 (2)	C25—C32	1.512 (3)
C6—C7	1.339 (2)	C26—C27	1.384 (3)
C7—C21	1.503 (2)	C26—H26	0.9300
C8—C9	1.468 (2)	C27—H27	0.9300
C8—H8	0.9300	C28—H28A	0.9600
C9—C14	1.395 (2)	C28—H28B	0.9600
C9—C10	1.397 (2)	C28—H28C	0.9600
C10—C11	1.385 (3)	C29—H29A	0.9600
C10—H10	0.9300	C29—H29B	0.9600
C11—C12	1.384 (3)	C29—H29C	0.9600
C11—H11	0.9300	C30—H30A	0.9600
C12—C13	1.390 (3)	C30—H30B	0.9600
C12—C31	1.509 (3)	C30—H30C	0.9600
C13—C14	1.385 (3)	C31—H31A	0.9600
C13—H13	0.9300	C31—H31B	0.9600
C14—H14	0.9300	C31—H31C	0.9600
C15—C20	1.344 (2)	C32—H32A	0.9600
C15—C16	1.492 (2)	C32—H32B	0.9600
C16—C17	1.537 (2)	C32—H32C	0.9600
C16—H16A	0.9700		
			
C15—O1—C6	116.89 (12)	C29—C17—C16	109.18 (16)
C28—N1—C1	115.90 (15)	C18—C17—C16	107.11 (15)
C28—N1—C4	115.93 (15)	C30—C17—C16	111.09 (17)
C1—N1—C4	102.00 (13)	C19—C18—C17	114.13 (16)
N1—C1—C7	111.86 (14)	C19—C18—H18A	108.7
N1—C1—C2	100.92 (14)	C17—C18—H18A	108.7
C7—C1—C2	110.16 (15)	C19—C18—H18B	108.7
N1—C1—H1	111.2	C17—C18—H18B	108.7
C7—C1—H1	111.2	H18A—C18—H18B	107.6
C2—C1—H1	111.2	O2—C19—C20	120.85 (17)
C1—C2—C3	103.11 (14)	O2—C19—C18	121.77 (17)
C1—C2—H2A	111.1	C20—C19—C18	117.30 (15)
C3—C2—H2A	111.1	C15—C20—C19	118.89 (15)
C1—C2—H2B	111.1	C15—C20—C21	122.19 (15)
C3—C2—H2B	111.1	C19—C20—C21	118.89 (14)
H2A—C2—H2B	109.1	C7—C21—C20	108.68 (13)
C4—C3—C2	104.38 (14)	C7—C21—C22	110.88 (15)
C4—C3—H3A	110.9	C20—C21—C22	111.55 (13)
C2—C3—H3A	110.9	C7—C21—H21	108.6
C4—C3—H3B	110.9	C20—C21—H21	108.6
C2—C3—H3B	110.9	C22—C21—H21	108.6
H3A—C3—H3B	108.9	C27—C22—C23	117.53 (18)
N1—C4—C5	112.42 (13)	C27—C22—C21	120.54 (16)
N1—C4—C3	100.68 (13)	C23—C22—C21	121.92 (17)
C5—C4—C3	111.48 (15)	C22—C23—C24	120.2 (2)
N1—C4—H4	110.6	C22—C23—H23	119.9
C5—C4—H4	110.6	C24—C23—H23	119.9
C3—C4—H4	110.6	C25—C24—C23	121.7 (2)
C8—C5—C6	122.22 (15)	C25—C24—H24	119.1
C8—C5—C4	125.27 (15)	C23—C24—H24	119.1
C6—C5—C4	112.48 (13)	C24—C25—C26	117.7 (2)
C7—C6—O1	122.44 (15)	C24—C25—C32	121.4 (2)
C7—C6—C5	122.87 (15)	C26—C25—C32	120.9 (3)
O1—C6—C5	114.63 (13)	C25—C26—C27	121.1 (2)
C6—C7—C21	123.02 (15)	C25—C26—H26	119.4
C6—C7—C1	117.77 (15)	C27—C26—H26	119.4
C21—C7—C1	119.21 (14)	C22—C27—C26	121.7 (2)
C5—C8—C9	127.43 (16)	C22—C27—H27	119.2
C5—C8—H8	116.3	C26—C27—H27	119.2
C9—C8—H8	116.3	N1—C28—H28A	109.5
C14—C9—C10	116.96 (15)	N1—C28—H28B	109.5
C14—C9—C8	123.43 (15)	H28A—C28—H28B	109.5
C10—C9—C8	119.55 (15)	N1—C28—H28C	109.5
C11—C10—C9	121.39 (16)	H28A—C28—H28C	109.5
C11—C10—H10	119.3	H28B—C28—H28C	109.5
C9—C10—H10	119.3	C17—C29—H29A	109.5
C10—C11—C12	121.46 (16)	C17—C29—H29B	109.5
C10—C11—H11	119.3	H29A—C29—H29B	109.5
C12—C11—H11	119.3	C17—C29—H29C	109.5
C11—C12—C13	117.25 (17)	H29A—C29—H29C	109.5
C11—C12—C31	121.57 (17)	H29B—C29—H29C	109.5
C13—C12—C31	121.17 (18)	C17—C30—H30A	109.5
C14—C13—C12	121.78 (17)	C17—C30—H30B	109.5
C14—C13—H13	119.1	H30A—C30—H30B	109.5
C12—C13—H13	119.1	C17—C30—H30C	109.5
C13—C14—C9	121.03 (16)	H30A—C30—H30C	109.5
C13—C14—H14	119.5	H30B—C30—H30C	109.5
C9—C14—H14	119.5	C12—C31—H31A	109.5
C20—C15—O1	123.55 (15)	C12—C31—H31B	109.5
C20—C15—C16	125.05 (15)	H31A—C31—H31B	109.5
O1—C15—C16	111.39 (13)	C12—C31—H31C	109.5
C15—C16—C17	112.91 (14)	H31A—C31—H31C	109.5
C15—C16—H16A	109.0	H31B—C31—H31C	109.5
C17—C16—H16A	109.0	C25—C32—H32A	109.5
C15—C16—H16B	109.0	C25—C32—H32B	109.5
C17—C16—H16B	109.0	H32A—C32—H32B	109.5
H16A—C16—H16B	107.8	C25—C32—H32C	109.5
C29—C17—C18	110.96 (18)	H32A—C32—H32C	109.5
C29—C17—C30	109.03 (18)	H32B—C32—H32C	109.5
C18—C17—C30	109.47 (16)		
			
C28—N1—C1—C7	62.30 (19)	C8—C9—C14—C13	−178.88 (19)
C4—N1—C1—C7	−64.57 (16)	C6—O1—C15—C20	7.7 (2)
C28—N1—C1—C2	179.41 (15)	C6—O1—C15—C16	−171.12 (14)
C4—N1—C1—C2	52.53 (15)	C20—C15—C16—C17	17.0 (3)
N1—C1—C2—C3	−32.38 (17)	O1—C15—C16—C17	−164.20 (15)
C7—C1—C2—C3	85.97 (17)	C15—C16—C17—C29	−167.58 (18)
C1—C2—C3—C4	2.13 (18)	C15—C16—C17—C18	−47.4 (2)
C28—N1—C4—C5	−58.9 (2)	C15—C16—C17—C30	72.1 (2)
C1—N1—C4—C5	67.99 (16)	C29—C17—C18—C19	174.65 (17)
C28—N1—C4—C3	−177.62 (16)	C30—C17—C18—C19	−65.0 (2)
C1—N1—C4—C3	−50.76 (15)	C16—C17—C18—C19	55.6 (2)
C2—C3—C4—N1	28.68 (17)	C17—C18—C19—O2	150.97 (18)
C2—C3—C4—C5	−90.75 (17)	C17—C18—C19—C20	−32.2 (2)
N1—C4—C5—C8	146.10 (17)	O1—C15—C20—C19	−168.96 (15)
C3—C4—C5—C8	−101.7 (2)	C16—C15—C20—C19	9.7 (3)
N1—C4—C5—C6	−36.0 (2)	O1—C15—C20—C21	8.9 (3)
C3—C4—C5—C6	76.16 (18)	C16—C15—C20—C21	−172.43 (16)
C15—O1—C6—C7	−12.9 (2)	O2—C19—C20—C15	174.93 (17)
C15—O1—C6—C5	164.29 (14)	C18—C19—C20—C15	−1.9 (2)
C8—C5—C6—C7	175.52 (17)	O2—C19—C20—C21	−3.0 (2)
C4—C5—C6—C7	−2.4 (2)	C18—C19—C20—C21	−179.88 (15)
C8—C5—C6—O1	−1.7 (2)	C6—C7—C21—C20	13.3 (2)
C4—C5—C6—O1	−179.63 (14)	C1—C7—C21—C20	−167.26 (14)
O1—C6—C7—C21	1.4 (3)	C6—C7—C21—C22	−109.67 (18)
C5—C6—C7—C21	−175.61 (15)	C1—C7—C21—C22	69.79 (19)
O1—C6—C7—C1	−178.09 (15)	C15—C20—C21—C7	−18.3 (2)
C5—C6—C7—C1	4.9 (3)	C19—C20—C21—C7	159.56 (14)
N1—C1—C7—C6	30.9 (2)	C15—C20—C21—C22	104.24 (18)
C2—C1—C7—C6	−80.5 (2)	C19—C20—C21—C22	−77.90 (19)
N1—C1—C7—C21	−148.60 (15)	C7—C21—C22—C27	61.8 (2)
C2—C1—C7—C21	100.01 (17)	C20—C21—C22—C27	−59.5 (2)
C6—C5—C8—C9	−174.93 (16)	C7—C21—C22—C23	−117.10 (19)
C4—C5—C8—C9	2.7 (3)	C20—C21—C22—C23	121.62 (19)
C5—C8—C9—C14	33.7 (3)	C27—C22—C23—C24	0.7 (3)
C5—C8—C9—C10	−149.18 (19)	C21—C22—C23—C24	179.61 (18)
C14—C9—C10—C11	−4.0 (3)	C22—C23—C24—C25	0.4 (3)
C8—C9—C10—C11	178.71 (17)	C23—C24—C25—C26	−1.2 (3)
C9—C10—C11—C12	1.5 (3)	C23—C24—C25—C32	177.8 (2)
C10—C11—C12—C13	1.1 (3)	C24—C25—C26—C27	0.8 (4)
C10—C11—C12—C31	−179.5 (2)	C32—C25—C26—C27	−178.1 (2)
C11—C12—C13—C14	−1.2 (3)	C23—C22—C27—C26	−1.0 (3)
C31—C12—C13—C14	179.5 (2)	C21—C22—C27—C26	−179.9 (2)
C12—C13—C14—C9	−1.4 (3)	C25—C26—C27—C22	0.2 (4)
C10—C9—C14—C13	3.9 (3)		

### (12E)-12-(4-Benzylidene)-7,7,16-trimethyl-3-(4-methylphenyl)-1-oxa-16-azatetracyclo[11.2.1.02,11.04,9]hexadeca-2(11),4(9)-dien-5-one (I) Hydrogen-bond geometry (Å, º)

*Cg* is the centroid of the C22–C27 ring.

**Table:**

*D*—H···*A*	*D*—H	H···*A*	*D*···*A*	*D*—H···*A*
C8—H8···O1	0.93	2.37	2.766 (2)	105
C14—H14···O2^i^	0.93	2.59	3.285 (2)	131
C3—H3*B*···*Cg*^i^	0.97	2.88	3.795 (2)	152

Symmetry code: (i) *x*−1/2, −*y*+3/2, −*z*.

### (12E)-12-(4-Bromobenzylidene)-73-(4-bromophenyl)-,7,16-trimethyl-10-oxa-16-azatetracyclo[11.2.1.02,11.04,9]hexadeca-2(11),4(9)-dien-5-one (II) Crystal data

**Table:**

C_30_H_29_Br_2_NO_2_	*F*(000) = 1208
*M**_r_* = 595.36	*D*_x_ = 1.491 Mg m^−^^3^
Monoclinic, *P*2_1_/*n*	Mo *K*α radiation, λ = 0.71073 Å
*a* = 16.051 (3) Å	Cell parameters from 102112 reflections
*b* = 9.7793 (17) Å	θ = 3.5–27.5°
*c* = 17.005 (3) Å	µ = 3.08 mm^−^^1^
β = 96.312 (5)°	*T* = 293 K
*V* = 2653.1 (8) Å^3^	Block, colourless
*Z* = 4	0.20 × 0.17 × 0.15 mm

### (12E)-12-(4-Bromobenzylidene)-73-(4-bromophenyl)-,7,16-trimethyl-10-oxa-16-azatetracyclo[11.2.1.02,11.04,9]hexadeca-2(11),4(9)-dien-5-one (II) Data collection

**Table:**

Bruker SMART APEX CCD area-detector diffractometer	*R*_int_ = 0.091
Radiation source: fine-focus sealed tube	θ_max_ = 28.3°, θ_min_ = 3.3°
ω and φ scans	*h* = −21→21
133143 measured reflections	*k* = −13→13
6556 independent reflections	*l* = −22→22
4933 reflections with *I* > 2σ(*I*)	

### (12E)-12-(4-Bromobenzylidene)-73-(4-bromophenyl)-,7,16-trimethyl-10-oxa-16-azatetracyclo[11.2.1.02,11.04,9]hexadeca-2(11),4(9)-dien-5-one (II) Refinement

**Table:**

Refinement on *F*^2^	0 restraints
Least-squares matrix: full	Hydrogen site location: inferred from neighbouring sites
*R*[*F*^2^ > 2σ(*F*^2^)] = 0.050	H-atom parameters constrained
*wR*(*F*^2^) = 0.141	*w* = 1/[σ^2^(*F*_o_^2^) + (0.0695*P*)^2^ + 2.235*P*] where *P* = (*F*_o_^2^ + 2*F*_c_^2^)/3
*S* = 1.09	(Δ/σ)_max_ = 0.004
6556 reflections	Δρ_max_ = 0.54 e Å^−^^3^
316 parameters	Δρ_min_ = −0.71 e Å^−^^3^

### (12E)-12-(4-Bromobenzylidene)-73-(4-bromophenyl)-,7,16-trimethyl-10-oxa-16-azatetracyclo[11.2.1.02,11.04,9]hexadeca-2(11),4(9)-dien-5-one (II) Special details

**Table:**

Geometry. All esds (except the esd in the dihedral angle between two l.s. planes) are estimated using the full covariance matrix. The cell esds are taken into account individually in the estimation of esds in distances, angles and torsion angles; correlations between esds in cell parameters are only used when they are defined by crystal symmetry. An approximate (isotropic) treatment of cell esds is used for estimating esds involving l.s. planes.

### (12E)-12-(4-Bromobenzylidene)-73-(4-bromophenyl)-,7,16-trimethyl-10-oxa-16-azatetracyclo[11.2.1.02,11.04,9]hexadeca-2(11),4(9)-dien-5-one (II) Fractional atomic coordinates and isotropic or equivalent isotropic displacement parameters (Å^2^)

**Table:**

	*x*	*y*	*z*	*U*_iso_*/*U*_eq_	
Br1	1.13657 (3)	1.48892 (4)	−0.08618 (3)	0.07424 (18)	
Br2	0.33512 (2)	0.92555 (5)	0.21664 (2)	0.05914 (15)	
O1	0.74355 (12)	0.8697 (2)	−0.04204 (11)	0.0324 (4)	
O2	0.60743 (18)	0.5052 (3)	0.07177 (15)	0.0552 (7)	
N1	0.77700 (15)	1.1142 (3)	0.15803 (14)	0.0350 (5)	
C1	0.75767 (19)	0.9713 (3)	0.17268 (16)	0.0349 (6)	
H1	0.715499	0.963908	0.209986	0.042*	
C2	0.8429 (2)	0.9194 (4)	0.21006 (17)	0.0429 (7)	
H2A	0.848064	0.821331	0.203353	0.052*	
H2B	0.851861	0.941265	0.265987	0.052*	
C3	0.90510 (19)	0.9981 (3)	0.16300 (17)	0.0367 (6)	
H3A	0.947246	1.044848	0.198476	0.044*	
H3B	0.932793	0.936504	0.129481	0.044*	
C4	0.84906 (17)	1.1019 (3)	0.11262 (16)	0.0299 (6)	
H4	0.877685	1.190003	0.109710	0.036*	
C5	0.82292 (16)	1.0451 (3)	0.03006 (16)	0.0278 (5)	
C6	0.76206 (16)	0.9339 (3)	0.03146 (15)	0.0277 (5)	
C7	0.73047 (16)	0.8929 (3)	0.09691 (15)	0.0280 (5)	
C8	0.85228 (18)	1.0867 (3)	−0.03686 (16)	0.0319 (6)	
H8	0.827454	1.047826	−0.083593	0.038*	
C9	0.91934 (18)	1.1868 (3)	−0.04467 (17)	0.0327 (6)	
C10	0.9139 (2)	1.2727 (4)	−0.11047 (19)	0.0451 (8)	
H10	0.866585	1.268859	−0.147241	0.054*	
C11	0.9776 (2)	1.3637 (4)	−0.1222 (2)	0.0529 (9)	
H11	0.972788	1.420918	−0.166140	0.064*	
C12	1.0477 (2)	1.3684 (3)	−0.0684 (2)	0.0443 (8)	
C13	1.05595 (19)	1.2852 (3)	−0.0033 (2)	0.0445 (7)	
H13	1.103820	1.289674	0.032729	0.053*	
C14	0.99204 (18)	1.1939 (3)	0.00834 (18)	0.0385 (7)	
H14	0.997839	1.136580	0.052242	0.046*	
C15	0.69997 (16)	0.7497 (3)	−0.04300 (15)	0.0280 (5)	
C16	0.69721 (19)	0.6819 (3)	−0.12194 (16)	0.0370 (7)	
H16A	0.749908	0.634979	−0.125406	0.044*	
H16B	0.691302	0.751430	−0.162876	0.044*	
C17	0.6257 (2)	0.5797 (3)	−0.13722 (18)	0.0415 (7)	
C18	0.6246 (3)	0.4881 (4)	−0.0646 (2)	0.0509 (9)	
H18A	0.573307	0.434798	−0.070523	0.061*	
H18B	0.671080	0.424447	−0.063439	0.061*	
C19	0.63029 (19)	0.5615 (3)	0.01397 (18)	0.0367 (6)	
C20	0.66752 (16)	0.6980 (3)	0.01977 (16)	0.0283 (5)	
C21	0.67489 (16)	0.7691 (3)	0.09920 (15)	0.0274 (5)	
H21	0.702592	0.705638	0.138323	0.033*	
C22	0.59016 (16)	0.8069 (3)	0.12632 (15)	0.0276 (5)	
C23	0.57771 (17)	0.7910 (3)	0.20543 (16)	0.0332 (6)	
H23	0.620545	0.755204	0.240721	0.040*	
C24	0.50238 (18)	0.8278 (3)	0.23264 (17)	0.0363 (6)	
H24	0.494637	0.817617	0.285727	0.044*	
C25	0.43931 (17)	0.8796 (3)	0.17940 (18)	0.0354 (6)	
C26	0.44916 (19)	0.8963 (4)	0.10059 (18)	0.0412 (7)	
H26	0.405744	0.930913	0.065491	0.049*	
C27	0.52543 (19)	0.8603 (3)	0.07442 (17)	0.0382 (7)	
H27	0.533120	0.872186	0.021438	0.046*	
C28	0.7072 (2)	1.1975 (4)	0.1221 (2)	0.0519 (9)	
H28A	0.726219	1.289261	0.114943	0.078*	
H28B	0.686260	1.159519	0.071727	0.078*	
H28C	0.663230	1.198414	0.156123	0.078*	
C29	0.6372 (3)	0.4933 (4)	−0.2101 (2)	0.0601 (11)	
H29A	0.591792	0.429284	−0.219197	0.090*	
H29B	0.637705	0.551816	−0.255393	0.090*	
H29C	0.689284	0.444488	−0.201590	0.090*	
C30	0.5420 (2)	0.6584 (5)	−0.1527 (2)	0.0626 (11)	
H30A	0.496677	0.594438	−0.162336	0.094*	
H30B	0.533914	0.713077	−0.107253	0.094*	
H30C	0.543537	0.716471	−0.197980	0.094*	

### (12E)-12-(4-Bromobenzylidene)-73-(4-bromophenyl)-,7,16-trimethyl-10-oxa-16-azatetracyclo[11.2.1.02,11.04,9]hexadeca-2(11),4(9)-dien-5-one (II) Atomic displacement parameters (Å^2^)

**Table:**

	*U*^11^	*U*^22^	*U*^33^	*U*^12^	*U*^13^	*U*^23^
Br1	0.0454 (2)	0.0471 (2)	0.1313 (4)	−0.01677 (16)	0.0145 (2)	0.0214 (2)
Br2	0.03657 (19)	0.0783 (3)	0.0654 (3)	0.01380 (17)	0.01814 (16)	0.00151 (19)
O1	0.0402 (11)	0.0323 (10)	0.0253 (9)	−0.0166 (8)	0.0059 (8)	−0.0024 (8)
O2	0.0722 (17)	0.0448 (14)	0.0517 (14)	−0.0212 (12)	0.0207 (13)	0.0075 (11)
N1	0.0362 (13)	0.0354 (13)	0.0337 (12)	−0.0015 (10)	0.0056 (10)	−0.0114 (10)
C1	0.0394 (15)	0.0426 (16)	0.0234 (13)	−0.0106 (13)	0.0066 (11)	−0.0059 (12)
C2	0.0471 (18)	0.0498 (19)	0.0301 (14)	−0.0089 (14)	−0.0039 (13)	0.0040 (13)
C3	0.0332 (14)	0.0429 (17)	0.0324 (14)	−0.0025 (12)	−0.0036 (11)	−0.0013 (12)
C4	0.0300 (13)	0.0301 (13)	0.0294 (13)	−0.0059 (11)	0.0018 (10)	−0.0052 (11)
C5	0.0275 (12)	0.0268 (13)	0.0286 (13)	−0.0040 (10)	0.0007 (10)	−0.0023 (10)
C6	0.0287 (12)	0.0276 (13)	0.0260 (12)	−0.0067 (10)	0.0002 (10)	−0.0020 (10)
C7	0.0260 (12)	0.0318 (13)	0.0262 (12)	−0.0039 (10)	0.0036 (10)	−0.0035 (11)
C8	0.0342 (14)	0.0321 (14)	0.0289 (13)	−0.0085 (11)	0.0014 (11)	−0.0017 (11)
C9	0.0354 (14)	0.0273 (13)	0.0360 (14)	−0.0064 (11)	0.0064 (11)	−0.0034 (11)
C10	0.0436 (17)	0.0460 (18)	0.0444 (17)	−0.0138 (14)	−0.0003 (14)	0.0095 (14)
C11	0.055 (2)	0.0427 (19)	0.061 (2)	−0.0121 (16)	0.0045 (17)	0.0216 (16)
C12	0.0358 (15)	0.0281 (15)	0.070 (2)	−0.0075 (12)	0.0128 (15)	0.0017 (15)
C13	0.0317 (15)	0.0413 (17)	0.060 (2)	−0.0050 (13)	0.0021 (14)	−0.0040 (15)
C14	0.0361 (15)	0.0357 (16)	0.0439 (16)	−0.0044 (12)	0.0054 (13)	0.0050 (13)
C15	0.0258 (12)	0.0285 (13)	0.0300 (13)	−0.0077 (10)	0.0035 (10)	−0.0024 (11)
C16	0.0402 (15)	0.0423 (16)	0.0303 (14)	−0.0176 (13)	0.0123 (12)	−0.0092 (12)
C17	0.0429 (16)	0.0484 (18)	0.0340 (15)	−0.0232 (14)	0.0079 (13)	−0.0104 (13)
C18	0.065 (2)	0.0374 (18)	0.0516 (19)	−0.0236 (16)	0.0127 (17)	−0.0066 (15)
C19	0.0369 (15)	0.0321 (15)	0.0422 (16)	−0.0084 (12)	0.0095 (12)	0.0013 (13)
C20	0.0267 (12)	0.0274 (13)	0.0317 (13)	−0.0044 (10)	0.0075 (10)	0.0009 (11)
C21	0.0264 (12)	0.0304 (13)	0.0256 (12)	−0.0019 (10)	0.0036 (10)	0.0043 (10)
C22	0.0267 (12)	0.0273 (13)	0.0294 (13)	−0.0032 (10)	0.0052 (10)	0.0044 (10)
C23	0.0315 (13)	0.0398 (15)	0.0288 (13)	0.0026 (12)	0.0048 (11)	0.0076 (12)
C24	0.0373 (15)	0.0426 (16)	0.0305 (14)	0.0011 (13)	0.0107 (12)	0.0044 (12)
C25	0.0285 (13)	0.0360 (15)	0.0430 (16)	0.0017 (11)	0.0093 (12)	0.0006 (12)
C26	0.0333 (15)	0.0524 (19)	0.0374 (15)	0.0102 (13)	0.0014 (12)	0.0091 (14)
C27	0.0381 (15)	0.0481 (18)	0.0287 (14)	0.0040 (13)	0.0052 (12)	0.0097 (13)
C28	0.0456 (18)	0.049 (2)	0.061 (2)	0.0079 (16)	0.0042 (16)	−0.0160 (17)
C29	0.074 (3)	0.059 (2)	0.049 (2)	−0.037 (2)	0.0153 (18)	−0.0224 (17)
C30	0.0434 (19)	0.088 (3)	0.054 (2)	−0.014 (2)	−0.0024 (16)	−0.001 (2)

### (12E)-12-(4-Bromobenzylidene)-73-(4-bromophenyl)-,7,16-trimethyl-10-oxa-16-azatetracyclo[11.2.1.02,11.04,9]hexadeca-2(11),4(9)-dien-5-one (II) Geometric parameters (Å, º)

**Table:**

Br1—C12	1.899 (3)	C15—C20	1.338 (4)
Br2—C25	1.906 (3)	C15—C16	1.493 (4)
O1—C15	1.365 (3)	C16—C17	1.524 (4)
O1—C6	1.401 (3)	C16—H16A	0.9700
O2—C19	1.218 (4)	C16—H16B	0.9700
N1—C1	1.458 (4)	C17—C29	1.528 (5)
N1—C4	1.464 (4)	C17—C18	1.528 (5)
N1—C28	1.464 (4)	C17—C30	1.544 (5)
C1—C7	1.522 (4)	C18—C19	1.510 (5)
C1—C2	1.531 (4)	C18—H18A	0.9700
C1—H1	0.9800	C18—H18B	0.9700
C2—C3	1.550 (4)	C19—C20	1.462 (4)
C2—H2A	0.9700	C20—C21	1.512 (4)
C2—H2B	0.9700	C21—C22	1.529 (4)
C3—C4	1.551 (4)	C21—H21	0.9800
C3—H3A	0.9700	C22—C27	1.389 (4)
C3—H3B	0.9700	C22—C23	1.390 (4)
C4—C5	1.525 (4)	C23—C24	1.389 (4)
C4—H4	0.9800	C23—H23	0.9300
C5—C8	1.342 (4)	C24—C25	1.378 (4)
C5—C6	1.464 (4)	C24—H24	0.9300
C6—C7	1.335 (4)	C25—C26	1.376 (4)
C7—C21	1.507 (4)	C26—C27	1.393 (4)
C8—C9	1.472 (4)	C26—H26	0.9300
C8—H8	0.9300	C27—H27	0.9300
C9—C10	1.394 (4)	C28—H28A	0.9600
C9—C14	1.396 (4)	C28—H28B	0.9600
C10—C11	1.386 (4)	C28—H28C	0.9600
C10—H10	0.9300	C29—H29A	0.9600
C11—C12	1.371 (5)	C29—H29B	0.9600
C11—H11	0.9300	C29—H29C	0.9600
C12—C13	1.368 (5)	C30—H30A	0.9600
C13—C14	1.391 (4)	C30—H30B	0.9600
C13—H13	0.9300	C30—H30C	0.9600
C14—H14	0.9300		
			
C15—O1—C6	117.0 (2)	C15—C16—H16B	109.0
C1—N1—C4	101.9 (2)	C17—C16—H16B	109.0
C1—N1—C28	115.9 (3)	H16A—C16—H16B	107.8
C4—N1—C28	116.0 (3)	C16—C17—C29	110.2 (3)
N1—C1—C7	112.6 (2)	C16—C17—C18	108.8 (3)
N1—C1—C2	101.0 (2)	C29—C17—C18	110.2 (3)
C7—C1—C2	110.1 (3)	C16—C17—C30	109.1 (3)
N1—C1—H1	110.9	C29—C17—C30	108.5 (3)
C7—C1—H1	110.9	C18—C17—C30	110.1 (3)
C2—C1—H1	110.9	C19—C18—C17	115.5 (3)
C1—C2—C3	102.6 (2)	C19—C18—H18A	108.4
C1—C2—H2A	111.3	C17—C18—H18A	108.4
C3—C2—H2A	111.3	C19—C18—H18B	108.4
C1—C2—H2B	111.3	C17—C18—H18B	108.4
C3—C2—H2B	111.3	H18A—C18—H18B	107.5
H2A—C2—H2B	109.2	O2—C19—C20	121.0 (3)
C4—C3—C2	104.1 (2)	O2—C19—C18	120.3 (3)
C4—C3—H3A	110.9	C20—C19—C18	118.6 (3)
C2—C3—H3A	110.9	C15—C20—C19	118.9 (2)
C4—C3—H3B	110.9	C15—C20—C21	122.7 (2)
C2—C3—H3B	110.9	C19—C20—C21	118.1 (2)
H3A—C3—H3B	109.0	C7—C21—C20	109.6 (2)
N1—C4—C5	111.2 (2)	C7—C21—C22	111.3 (2)
N1—C4—C3	101.9 (2)	C20—C21—C22	113.3 (2)
C5—C4—C3	110.7 (2)	C7—C21—H21	107.5
N1—C4—H4	110.9	C20—C21—H21	107.5
C5—C4—H4	110.9	C22—C21—H21	107.5
C3—C4—H4	110.9	C27—C22—C23	118.7 (3)
C8—C5—C6	122.4 (2)	C27—C22—C21	121.8 (2)
C8—C5—C4	125.7 (2)	C23—C22—C21	119.5 (2)
C6—C5—C4	111.9 (2)	C24—C23—C22	121.1 (3)
C7—C6—O1	123.1 (2)	C24—C23—H23	119.4
C7—C6—C5	123.5 (2)	C22—C23—H23	119.4
O1—C6—C5	113.2 (2)	C25—C24—C23	118.7 (3)
C6—C7—C21	122.9 (2)	C25—C24—H24	120.7
C6—C7—C1	117.0 (2)	C23—C24—H24	120.7
C21—C7—C1	119.9 (2)	C26—C25—C24	121.9 (3)
C5—C8—C9	127.3 (3)	C26—C25—Br2	119.6 (2)
C5—C8—H8	116.3	C24—C25—Br2	118.4 (2)
C9—C8—H8	116.3	C25—C26—C27	118.7 (3)
C10—C9—C14	117.5 (3)	C25—C26—H26	120.7
C10—C9—C8	119.4 (3)	C27—C26—H26	120.7
C14—C9—C8	122.9 (3)	C22—C27—C26	121.0 (3)
C11—C10—C9	121.4 (3)	C22—C27—H27	119.5
C11—C10—H10	119.3	C26—C27—H27	119.5
C9—C10—H10	119.3	N1—C28—H28A	109.5
C12—C11—C10	119.4 (3)	N1—C28—H28B	109.5
C12—C11—H11	120.3	H28A—C28—H28B	109.5
C10—C11—H11	120.3	N1—C28—H28C	109.5
C13—C12—C11	121.2 (3)	H28A—C28—H28C	109.5
C13—C12—Br1	119.4 (2)	H28B—C28—H28C	109.5
C11—C12—Br1	119.4 (3)	C17—C29—H29A	109.5
C12—C13—C14	119.4 (3)	C17—C29—H29B	109.5
C12—C13—H13	120.3	H29A—C29—H29B	109.5
C14—C13—H13	120.3	C17—C29—H29C	109.5
C13—C14—C9	121.1 (3)	H29A—C29—H29C	109.5
C13—C14—H14	119.4	H29B—C29—H29C	109.5
C9—C14—H14	119.4	C17—C30—H30A	109.5
C20—C15—O1	124.0 (2)	C17—C30—H30B	109.5
C20—C15—C16	125.0 (2)	H30A—C30—H30B	109.5
O1—C15—C16	110.9 (2)	C17—C30—H30C	109.5
C15—C16—C17	113.1 (2)	H30A—C30—H30C	109.5
C15—C16—H16A	109.0	H30B—C30—H30C	109.5
C17—C16—H16A	109.0		
			
C4—N1—C1—C7	−63.9 (3)	C8—C9—C14—C13	176.6 (3)
C28—N1—C1—C7	63.1 (3)	C6—O1—C15—C20	6.7 (4)
C4—N1—C1—C2	53.6 (2)	C6—O1—C15—C16	−170.6 (2)
C28—N1—C1—C2	−179.4 (3)	C20—C15—C16—C17	23.8 (4)
N1—C1—C2—C3	−36.9 (3)	O1—C15—C16—C17	−158.9 (3)
C7—C1—C2—C3	82.4 (3)	C15—C16—C17—C29	−168.1 (3)
C1—C2—C3—C4	8.1 (3)	C15—C16—C17—C18	−47.3 (4)
C1—N1—C4—C5	70.2 (3)	C15—C16—C17—C30	72.8 (3)
C28—N1—C4—C5	−56.7 (3)	C16—C17—C18—C19	47.9 (4)
C1—N1—C4—C3	−47.8 (2)	C29—C17—C18—C19	168.7 (3)
C28—N1—C4—C3	−174.7 (2)	C30—C17—C18—C19	−71.6 (4)
C2—C3—C4—N1	23.3 (3)	C17—C18—C19—O2	159.9 (3)
C2—C3—C4—C5	−95.1 (3)	C17—C18—C19—C20	−23.2 (5)
N1—C4—C5—C8	140.3 (3)	O1—C15—C20—C19	−173.2 (3)
C3—C4—C5—C8	−107.1 (3)	C16—C15—C20—C19	3.7 (4)
N1—C4—C5—C6	−41.3 (3)	O1—C15—C20—C21	0.8 (4)
C3—C4—C5—C6	71.2 (3)	C16—C15—C20—C21	177.8 (3)
C15—O1—C6—C7	−8.7 (4)	O2—C19—C20—C15	172.8 (3)
C15—O1—C6—C5	168.1 (2)	C18—C19—C20—C15	−4.1 (4)
C8—C5—C6—C7	−178.4 (3)	O2—C19—C20—C21	−1.5 (4)
C4—C5—C6—C7	3.2 (4)	C18—C19—C20—C21	−178.5 (3)
C8—C5—C6—O1	4.8 (4)	C6—C7—C21—C20	4.1 (4)
C4—C5—C6—O1	−173.6 (2)	C1—C7—C21—C20	−171.7 (2)
O1—C6—C7—C21	3.0 (4)	C6—C7—C21—C22	−122.0 (3)
C5—C6—C7—C21	−173.5 (2)	C1—C7—C21—C22	62.2 (3)
O1—C6—C7—C1	178.9 (2)	C15—C20—C21—C7	−6.0 (4)
C5—C6—C7—C1	2.4 (4)	C19—C20—C21—C7	168.1 (2)
N1—C1—C7—C6	29.9 (4)	C15—C20—C21—C22	118.9 (3)
C2—C1—C7—C6	−82.1 (3)	C19—C20—C21—C22	−67.0 (3)
N1—C1—C7—C21	−154.1 (2)	C7—C21—C22—C27	82.3 (3)
C2—C1—C7—C21	93.9 (3)	C20—C21—C22—C27	−41.7 (4)
C6—C5—C8—C9	−173.3 (3)	C7—C21—C22—C23	−96.0 (3)
C4—C5—C8—C9	4.9 (5)	C20—C21—C22—C23	140.0 (3)
C5—C8—C9—C10	−145.2 (3)	C27—C22—C23—C24	−0.1 (4)
C5—C8—C9—C14	39.5 (5)	C21—C22—C23—C24	178.2 (3)
C14—C9—C10—C11	−1.1 (5)	C22—C23—C24—C25	0.5 (5)
C8—C9—C10—C11	−176.7 (3)	C23—C24—C25—C26	−0.3 (5)
C9—C10—C11—C12	0.6 (6)	C23—C24—C25—Br2	178.4 (2)
C10—C11—C12—C13	−0.1 (6)	C24—C25—C26—C27	−0.4 (5)
C10—C11—C12—Br1	178.0 (3)	Br2—C25—C26—C27	−179.1 (3)
C11—C12—C13—C14	0.1 (5)	C23—C22—C27—C26	−0.6 (5)
Br1—C12—C13—C14	−178.0 (2)	C21—C22—C27—C26	−178.9 (3)
C12—C13—C14—C9	−0.7 (5)	C25—C26—C27—C22	0.8 (5)
C10—C9—C14—C13	1.2 (5)		

### (12E)-12-(4-Bromobenzylidene)-73-(4-bromophenyl)-,7,16-trimethyl-10-oxa-16-azatetracyclo[11.2.1.02,11.04,9]hexadeca-2(11),4(9)-dien-5-one (II) Hydrogen-bond geometry (Å, º)

*Cg* is the centroid of the C9–C14 ring.

**Table:**

*D*—H···*A*	*D*—H	H···*A*	*D*···*A*	*D*—H···*A*
C8—H8···O1	0.93	2.36	2.742 (3)	105
C30—H30*A*···O2^i^	0.96	2.58	3.306 (5)	132
C3—H3*B*···*Cg*^ii^	0.97	2.84	3.798 (3)	169

Symmetry codes: (i) −*x*+1, −*y*+1, −*z*; (ii) −*x*+2, −*y*+2, −*z*.
